# Elective freezing of embryos versus fresh embryo transfer in IVF: a multicentre randomized controlled trial in the UK (E-Freeze)

**DOI:** 10.1093/humrep/deab279

**Published:** 2022-01-06

**Authors:** Abha Maheshwari, Jennifer L Bell, Priya Bhide, Daniel Brison, Tim Child, Huey Yi Chong, Ying Cheong, Christina Cole, Arri Coomarasamy, Rachel Cutting, Pollyanna Hardy, Haitham Hamoda, Edmund Juszczak, Yacoub Khalaf, Jennifer J Kurinczuk, Stuart Lavery, Louise Linsell, Nick Macklon, Raj Mathur, Jyotsna Pundir, Nick Raine-Fenning, Madhurima Rajkohwa, Graham Scotland, Kayleigh Stanbury, Stephen Troup, Siladitya Bhattacharya

**Affiliations:** 1 Aberdeen Fertility Centre, NHS Grampian and University of Aberdeen, Aberdeen, UK; 2 National Perinatal Epidemiology Unit, Nuffield Department of Population Health, University of Oxford, Oxford, UK; 3 Assisted Conception Unit, Homerton University Hospital NHS Foundation Trust and Queen Mary University of London, London, UK; 4 Assisted Conception Unit, Manchester University NHS Foundation Trust, Manchester, UK; 5 Oxford Fertility, TFP, University of Oxford, UK; 6 Health Economics Research Unit, University of Aberdeen, Aberdeen, UK; 7 Complete Fertility, University of Southampton, Southampton, UK; 8 Department of Metabolomics, University of Birmingham, Birmingham, UK; 9 Human Embryology Fertilisation Authority, London, UK; 10 Assisted Conception Unit, King’s College Hospital, London, UK; 11 Nottingham Clinical Trials Unit, University of Nottingham, Nottingham, UK; 12 Assisted Conception Unit and Centre for Pre-implantation Genetic Diagnosis, Guy’s and St Thomas’ Hospital and King’s College London, London, UK; 13 Gynaecology, Imperial College London, London, UK; 14 London Women’s Clinic, London, UK; 15 University of Copenhagen, Denmark; 16 Assisted Conception Unit, St. Mary’s Hospital, Manchester, UK; 17 Assisted Conception Unit, St. Bartholomew’s Hospital and Queen Mary University of London, London, UK; 18 Nurture Fertility, UK; 19 CARE Fertility, Birmingham, UK; 20 Reproductive Science Consultancy, UK

**Keywords:** IVF / frozen embryo transfer / freeze all / healthy baby / cost-effectiveness / fresh embryo transfer / infertility / willingness to pay

## Abstract

**STUDY QUESTION:**

Does a policy of elective freezing of embryos, followed by frozen embryo transfer result in a higher healthy baby rate, after first embryo transfer, when compared with the current policy of transferring fresh embryos?

**SUMMARY ANSWER:**

This study, although limited by sample size, provides no evidence to support the adoption of a routine policy of elective freeze in preference to fresh embryo transfer in order to improve IVF effectiveness in obtaining a healthy baby.

**WHAT IS KNOWN ALREADY:**

The policy of freezing all embryos followed by frozen embryo transfer is associated with a higher live birth rate for high responders but a similar/lower live birth after first embryo transfer and cumulative live birth rate for normal responders. Frozen embryo transfer is associated with a lower risk of ovarian hyperstimulation syndrome (OHSS), preterm delivery and low birthweight babies but a higher risk of large babies and pre-eclampsia. There is also uncertainty about long-term outcomes, hence shifting to a policy of elective freezing for all remains controversial given the delay in treatment and extra costs involved in freezing all embryos.

**STUDY DESIGN, SIZE, DURATION:**

A pragmatic two-arm parallel randomized controlled trial (E-Freeze) was conducted across 18 clinics in the UK from 2016 to 2019. A total of 619 couples were randomized (309 to elective freeze/310 to fresh). The primary outcome was a healthy baby after first embryo transfer (term, singleton live birth with appropriate weight for gestation); secondary outcomes included OHSS, live birth, clinical pregnancy, pregnancy complications and cost-effectiveness.

**PARTICIPANTS/MATERIALS, SETTING, METHODS:**

Couples undergoing their first, second or third cycle of IVF/ICSI treatment, with at least three good quality embryos on Day 3 where the female partner was ≥18 and <42 years of age were eligible. Those using donor gametes, undergoing preimplantation genetic testing or planning to freeze all their embryos were excluded. IVF/ICSI treatment was carried out according to local protocols. Women were followed up for pregnancy outcome after first embryo transfer following randomization.

**MAIN RESULTS AND THE ROLE OF CHANCE:**

Of the 619 couples randomized, 307 and 309 couples in the elective freeze and fresh transfer arms, respectively, were included in the primary analysis. There was no evidence of a statistically significant difference in outcomes in the elective freeze group compared to the fresh embryo transfer group: healthy baby rate {20.3% (62/307) versus 24.4% (75/309); risk ratio (RR), 95% CI: 0.84, 0.62 to 1.15}; OHSS (3.6% versus 8.1%; RR, 99% CI: 0.44, 0.15 to 1.30); live birth rate (28.3% versus 34.3%; RR, 99% CI 0.83, 0.65 to 1.06); and miscarriage (14.3% versus 12.9%; RR, 99% CI: 1.09, 0.72 to 1.66). Adherence to allocation was poor in the elective freeze group. The elective freeze approach was more costly and was unlikely to be cost-effective in a UK National Health Service context.

**LIMITATIONS, REASONS FOR CAUTION:**

We have only reported on first embryo transfer after randomization; data on the cumulative live birth rate requires further follow-up. Planned target sample size was not obtained and the non-adherence to allocation rate was high among couples in the elective freeze arm owing to patient preference for fresh embryo transfer, but an analysis which took non-adherence into account showed similar results.

**WIDER IMPLICATIONS OF THE FINDINGS:**

Results from the E-Freeze trial do not lend support to the policy of electively freezing all for everyone, taking both efficacy, safety and costs considerations into account. This method should only be adopted if there is a definite clinical indication.

**STUDY FUNDING/COMPETING INTEREST(S):**

NIHR Health Technology Assessment programme (13/115/82). This research was funded by the National Institute for Health Research (NIHR) (NIHR unique award identifier) using UK aid from the UK Government to support global health research. The views expressed in this publication are those of the author(s) and not necessarily those of the NIHR or the UK Department of Health and Social Care. J.L.B., C.C., E.J., P.H., J.J.K., L.L. and G.S. report receipt of funding from NIHR, during the conduct of the study. J.L.B., E.J., P.H., K.S. and L.L. report receipt of funding from NIHR, during the conduct of the study and outside the submitted work. A.M. reports grants from NIHR personal fees from Merck Serono, personal fees for lectures from Merck Serono, Ferring and Cooks outside the submitted work; travel/meeting support from Ferring and Pharmasure and participation in a Ferring advisory board. S.B. reports receipt of royalties and licenses from Cambridge University Press, a board membership role for NHS Grampian and other financial or non-financial interests related to his roles as Editor-in-Chief of *Human Reproduction Open* and Editor and Contributing Author of *Reproductive Medicine* for the MRCOG, Cambridge University Press. D.B. reports grants from NIHR, during the conduct of the study; grants from European Commission, grants from Diabetes UK, grants from NIHR, grants from ESHRE, grants from MRC, outside the submitted work. Y.C. reports speaker fees from Merck Serono, and advisory board role for Merck Serono and shares in Complete Fertility. P.H. reports membership of the HTA Commissioning Committee. E.J. reports membership of the NHS England and NIHR Partnership Programme, membership of five Data Monitoring Committees (Chair of two), membership of six Trial Steering Committees (Chair of four), membership of the Northern Ireland Clinical Trials Unit Advisory Group and Chair of the board of Oxford Brain Health Clinical Trials Unit. R.M. reports consulting fees from Gedeon Richter, honorarium from Merck, support fees for attendance at educational events and conferences for Merck, Ferring, Bessins and Gedeon Richter, payments for participation on a Merck Safety or Advisory Board, Chair of the British Fertility Society and payments for an advisory role to the Human Fertilisation and Embryology Authority. G.S. reports travel and accommodation fees for attendance at a health economic advisory board from Merck KGaA, Darmstadt, Germany. N.R.-F. reports shares in Nurture Fertility. Other authors’ competing interests: none declared.

**TRIAL REGISTRATION NUMBER:**

ISRCTN: 61225414.

**TRIAL REGISTRATION DATE:**

29 December 2015.

**DATE OF FIRST PATIENT’S ENROLMENT:**

16 February 2016.

## Introduction

Infertility affects one in six couples in the UK (Oakley *et al.*, 2008) and the recommended treatment for those with prolonged unresolved infertility is IVF (https://www.nice.org.uk/guidance/cg156).

In 2018, the average live birth rate per embryo transferred in the UK was 23% (Human Embryology Fertilisation Authority (HFEA) https://www.hfea.gov.uk/about-us/publications/research-and-data/), and clinics and patients continue to explore ways of increasing success rates. Advances in freezing techniques have allowed the possibility of electively freezing all suitable embryos (elective freeze), avoiding replacing them as fresh embryos. It has been suggested that transfer of frozen–thawed embryos in a non-stimulated cycle is more conducive to early placentation and embryogenesis when compared with fresh IVF cycles.

Previous systematic reviews have shown poorer maternal and perinatal outcomes in pregnancies following IVF (Pandey *et al.*, 2012), particularly after fresh embryo transfer (Maheshwari *et al.*, 2012), compared to those in the general population. IVF is also associated with risk of ovarian hyperstimulation syndrome (OHSS), which can cause significant maternal morbidity and, rarely, mortality. It has been suggested that avoiding fresh embryo transfer by electively freezing embryos followed by frozen embryo transfer reduces the chance of OHSS (Devroey *et al.*, 2011), decreases maternal and perinatal risks (Maheshwari *et al.*, 2012) and improves pregnancy rates (Shapiro *et al.*, 2011a,b). Hence there have been suggestions that practice should change to elective freeze for all women, in preference to the current practice of fresh embryo transfer.

This led to a number of randomized trials across the world. Although trials on women at significant risk of OHSS suggest that an elective freeze strategy increases live birth rates per first embryo transfer (Chen *et al.*, 2016; Aflatoonian *et al.*, 2018), the evidence is less clear for others undergoing IVF. Most studies show no difference (Shi *et al.*, 2018; Vuong *et al.*, 2018; Stormlund *et al.*, 2020), while others show improvement (Wei *et al.*, 2019) in live birth after first embryo transfer, or reduction (Wong *et al.*, 2021) in cumulative live birth rates. Cumulative live birth rate over multiple embryo transfers may be reduced by a routine elective freeze policy, as per data from the HFEA (Smith *et al.*, 2019), whereas a recent Cochrane review showed no difference (Zaat *et al.*, 2021).

The Cochrane review (Zaat *et al.*, 2021) also suggested that an elective freeze approach may increase the hypertensive disorders of pregnancy, large for gestational age (LGA) babies and the birthweight of children. There was uncertainty about the risk of small for gestational age (SGA) babies, but the evidence was of low quality. Despite the continuing scientific debate on this subject, there has been an exponential rise in the adoption of an elective freeze approach. In the UK, fresh embryo transfers decreased by 11% between 2013 and 2018, while the numbers of frozen embryo transfer almost doubled over this period, accounting for 34% of all IVF cycles in 2018.

As events during pregnancy and birth have long-term implications it is important to consider not just live birth rate, but also the health of the baby at delivery before opting for an elective freeze policy in preference to fresh embryo transfer for all. Almost all trials on this topic have reported on live birth as the primary outcome, whereas the ultimate aim of fertility treatments is to have both a healthy mother and a healthy baby.

The primary objective of the E-Freeze trial reported here was to determine if a policy of electively freezing all suitable embryos, followed by frozen embryo transfer would result in a higher healthy baby rate following the first embryo transfer when compared with the current policy of transferring fresh embryos, where a healthy baby was defined as term singleton live birth with appropriate weight for gestation.

## Materials and methods

### Study design and participants

This was a non-blinded two-arm parallel-group multicentre pragmatic randomized controlled trial (RCT) conducted across 18 IVF clinics in the UK. The E-Freeze trial protocol was approved by the North of Scotland Research Ethics Service (NoSRES) Committee (Study Ref: 15/NS/0114). Local approval and site-specific assessments were obtained from each participating site.

### Participants

Women between 18 and 42 years of age, undergoing their first, second or third cycle of IVF, were eligible. At the outset of the trial, only first cycle patients were included. However, owing to low recruitment and after discussion with the funders, the inclusion criteria were expanded to incorporate second and third cycles as well. Exclusion criteria included use of donor gametes, pre-implantation genetic testing and a clinical indication for an elective freeze such as OHSS or fertility preservation. Women underwent controlled ovarian stimulation, egg retrieval, mixing of eggs and sperm, embryo culture, freezing and thawing of embryos following locally approved clinical and laboratory protocols.

### Randomization, allocation concealment and blinding

Randomization was performed on Day 3 following egg retrieval, in couples who fulfilled the final inclusion criteria of having at least three good quality embryos. Good quality embryos were defined as per nationally agreed criteria (Cutting *et al.*, 2008). Couples were randomized (1:1 allocation ratio) to either elective freeze or to fresh embryo transfer.

Randomization was performed using a 24/7 secure internet-based randomization system hosted by the University of Oxford. The randomization employed a probabilistic minimization algorithm to balance across the following factors: fertility clinic, female partner’s age at time of ovarian stimulation (<35 years/35 to <40 years/≥40 years), infertility (primary/secondary), self-reported duration of infertility (<12 months/12 to <24 months/24 to <36 months/36 to <48 months/48 to <60 months/≥60 months), method of insemination (IVF/ICSI or a combination of both) and number of previous egg collections (0/1/2 cycles) to account for first, second or third cycle. For each minimization stratum, the total number of existing participants in the same stratum as the new participant was calculated for each allocation. If the absolute difference between the totals was <3, the participant was allocated randomly to treatment A or B (with equal probability). If the absolute difference between the totals was >2, the participant was allocated to the allocation with the lowest total with probability 0.8.

Blinding of the allocated intervention was not possible because of the nature of the treatments, ethical considerations and statutory requirements of the regulatory body the HFEA.

### Interventions

In the intervention arm, all suitable embryos were frozen, while in the standard care arm women underwent fresh embryo transfer. Couples who were randomized to elective freeze were contacted within 3 working days post-randomization and arrangements made for frozen embryo transfer within 3 months of egg collection.

### Outcomes

The primary outcome was a healthy baby, defined as a live, singleton baby born at term (between 37 and 42 completed weeks of gestation) with an appropriate weight for gestation (weight between 10th and 90th centile for that gestation based on standardized charts) after first embryo transfer following randomization.

A pregnancy test was carried out in all randomized women 2 weeks after embryo transfer. All women who had a positive pregnancy test underwent a transvaginal ultrasound scan at 6–8 weeks of gestation in pregnancy to identify the presence of a gestational sac with a fetal heartbeat, signifying an ongoing pregnancy.

The secondary outcomes included measures of maternal safety during IVF (OHSS): clinical effectiveness (live birth rate and clinical pregnancy rate), complications of pregnancy and delivery (miscarriage rate, gestational diabetes, hypertensive disorders of pregnancy, antepartum haemorrhage, preterm delivery, mode of delivery, low birthweight, high birthweight, SGA, LGA and congenital anomalies) and cost-effectiveness (incremental cost per healthy baby and per live birth). Detailed definitions of each are in the published protocol (Maheshwari *et al.*, 2019). All outcomes are reported for first embryo transfer after randomization.

Women who had an ongoing pregnancy were contacted by their research nurse (by telephone) to record pregnancy events and outcomes at 12 and 28 weeks of gestation, and again approximately 6 weeks after delivery. Those who had a negative pregnancy test were not followed up any further as part of this trial.

### Economic evaluation

Health care resource use and pregnancy outcomes from randomization up to, and including, delivery were assessed using the trial electronic case report forms. Post-randomization IVF-related treatment costs were derived for the following categories: freezing of embryos, endometrial preparation, luteal support, and embryo transfer as well as thawing of frozen embryos, extra monitoring visits, blood tests and transvaginal ultrasound scans prior to frozen embryo transfer. Individual patient resource use data were valued from a National Health Service (NHS) perspective using unit costs derived from UK national sources (Curtis and Burns, 2019; Department of Health and Social Care, 2020). Costs were expressed in 2018/2019 pounds sterling. Full details of the economic analysis and modelling to extrapolate longer-term cost-effectiveness will be published elsewhere. The main within trial cost-effectiveness findings are presented in this paper.

### Statistical analysis

In order to achieve 90% power at a two-sided 5% level of statistical significance, 1086 women (543 per group) were required to show an absolute risk difference in the primary outcome of 8% (from 17% to 25%), between fresh embryo transfer and elective freeze strategy following first embryo transfer. A difference of 8% was considered to be clinically important by an expert panel of clinicians and scientists in order to recommend a change in routine clinical practice, considering the extra time, effort and cost involved in electively freezing all suitable embryos in preference of fresh embryo transfer.

A detailed statistical analysis plan has been published (Bell *et al.*, 2020). The primary analysis for all primary and secondary outcomes was by intention to treat (ITT). Secondary analyses were performed to include the clinically relevant denominators such as: per total number of women with a positive pregnancy test after embryo transfer, for miscarriage; per total number of pregnant women with an ongoing pregnancy resulting in delivery, for pregnancy complications; and per total number of babies born, for birthweight and congenital anomalies. For neonatal secondary outcomes, the unit of analysis in the ITT analysis was the mother and in cases of multiple pregnancy where the infants’ outcomes differed, the worst outcome was reported. In this article, results are reported per clinically relevant denominator.

Risk ratios (RRs) and CIs were calculated using a Poisson regression model with a robust variance estimator. Analyses were adjusted for all minimization factors, where technically possible. Adjusted and unadjusted RRs are presented, with the primary inference based on the adjusted estimates. Linear regression was used for normally distributed continuous outcomes and quantile regression for skewed continuous outcomes.

Pre-specified subgroup analyses for the primary outcome were: age (<35, ≥35 to <40 and ≥40 years); fertility clinic; cleavage versus blastocyst embryo transfer; single versus multiple embryo transfer; and number of previous embryo transfers.

For the primary outcome, 95% CIs were used for all analyses, and for secondary outcomes, 99% CIs to allow cautious interpretation of the results owing to the multiple number of hypothesis tests performed.

Further pre-specified analyses were carried out for the primary outcome only: complier-average causal effect analysis; per-protocol (restricted to those who complied with the allocated intervention), and as-treated (grouping couples according to allocation actually received).

For the within-trial cost-effectiveness analysis, generalized linear regression models with adjustment for design covariates were used to estimate mean differences in costs and effects by ITT. The incremental treatment cost (inclusive of OHSS costs) per additional healthy baby and per additional live birth per first embryo transfer was estimated as the measure of cost-effectiveness.

Non-parametric bootstrapping (1000 iterations) was used to characterize uncertainty surrounding the joint difference in costs and effects, and to determine the probability of the freeze-all strategy being cost-effective at different thresholds of willingness to pay (WTP) per healthy baby and per live birth following the first embryo transfer. Sensitivity analysis was conducted around the unit costs applied to transvaginal ultrasound scans as part of monitoring for frozen embryo transfer, and the inclusion of antenatal and delivery care costs. Analyses were performed using Stata version 15 (StataCorp, TX, USA).

## Results

**Figure 1.**
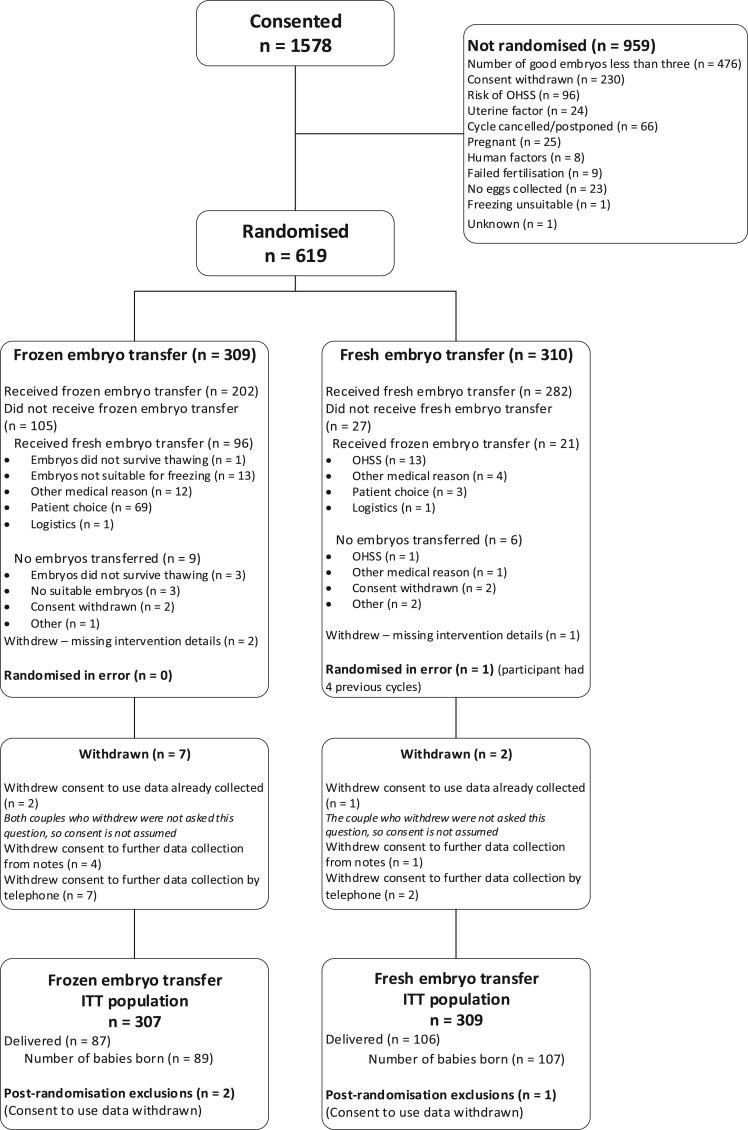
**Flow of participants in a randomized controlled trial (E-Freeze) of elective freezing of embryos versus fresh embryo transfer in IVF.** ITT, intention to treat; OHSS, ovarian hyperstimulation syndrome.

Between 16 February 2016 and 30 April 2019, 1578 couples consented to participate in the trial, of whom 619 were randomized: 309 to freeze-all and 310 to fresh embryo transfer. Most cases that did not progress to randomization (n = 959, 61%) were because of non-availability of three good quality embryos (n = 476, Fig.  1). Of those randomized, 117 (19%) did not adhere to their allocated intervention.

Recruitment was continually below expectation despite an in-built internal pilot and multiple strategies used to boost up recruitment. On 9 November 2018, the Data Monitoring Committee (DMC) recommended to the Trial Steering Committee (TSC) that the trial should be halted, owing to the shortfall in recruitment and the high level of non-adherence in the elective freeze group. Following the recommendation, a joint meeting of the TSC and DMC was convened on 17 January 2019, with an independent chair, to agree scenarios for a monitoring meeting with the National Institute for Health Research, Health Technology Assessment. After the monitoring meeting on 29 January 2019, it was agreed that the trial would stop recruitment on 30 April 2019 as it was felt that continuing the trial beyond then would yield no further benefit and lead to research wastage.

The ITT population included 307 couples in the elective freeze and 309 in the fresh embryo transfer arm, as three women withdrew consent for use of their data. Of 307 women randomized to elective freeze, 96 received fresh embryos (31%); non-adherence to the allocated intervention was much lower (n = 21, 7%) in the fresh embryo transfer arm. Personal choice accounted for 72% cases of non-adherence in the elective freeze arm, followed by 13% for medical reasons.

The two randomized groups were similar in terms of baseline characteristics (Table  I). The mean age of the women was 35 years with 95% of women under the age of 40 years, and 50% under the age of 35 years. Most women (78%) had primary infertility and a high proportion (41%) had unexplained infertility. Median (interquartile range (IQR)) duration of infertility for both arms was 36 months (IQR: 24–48 months).

**Table I:** Demographic and clinical characteristics of participants in a randomized controlled trial (E-Freeze) of elective freezing of embryos versus fresh embryo transfer in IVF.

	Frozen embryo transfer (n = 307)	Fresh embryo transfer (n = 309)
**At trial entry**		
Woman’s age at ovarian stimulation (years)*	34.7 (3.8)	34.6 (3.6)
**Non-smoker**	276 (89.9%)	282 (91.3%)
**Woman’s BMI (kg/m^2^)^†^**	24.1 (3.4)	24.1 (3.2)
Primary infertility*	237 (77.2%)	241 (78.0%)
**Primary cause of infertility**		
Ovulatory	40 (13.0%)	32 (10.4%)
Tubal	29 (9.4%)	27 (8.7%)
Endometriosis	13 (4.2%)	11 (3.6%)
Unexplained	119 (38.8%)	131 (42.4%)
Male	102 (33.2%)	102 (33.0%)
Other	4 (1.3%)	6 (1.9%)
Duration of infertility (months)*	36 (24 to 48)	36 (24 to 48)
**Total stimulation dose of FSH (IU)**	2539.8 (1256.6)	2543.2 (1259.2)
**Total number of eggs collected**	12 (9 to 16)	12 (9 to 17)
Method of insemination—IVF*	158 (51.5%)	159 (51.5%)
**Good quality embryos on Day 3**	5 (3 to 7)	5 (4 to 8)
No previous egg collections*	284 (92.5%)	286 (92.6%)

**During treatment**		
**Received embryo transfer**	298	303
Stage of embryo at transfer—blastocyst*	282/298 (94.6%)	282/303 (93.1%)
**Single embryo transfer**	249/298 (83.6%)	247/303 (81.5%)
**Number of remaining frozen embryos after transfer**		
0	68 (22.8%)	61 (20.8%)
1	46 (15.4%)	52 (17.2%)
2	55 (18.5%)	55 (18.2%)
≥3	129 (43.3%)	135 (44.6%)
**Received frozen transfer**	202	21
**Method of embryo freezing—vitrification**	178/202 (88.1%)	20/21 (95.2%)
**Method of endometrial preparation for frozen transfer^††^**		
Natural cycle	10/202 (5.0%)	6/21 (28.6%)
Hormone mediated cycle	191/202 (94.6%)	15/21 (71.4%)

Data are presented as mean (SD), median (IQR), N or n/N.

* Minimization factor.

† One observation missing in each arm.

†† One woman had other method used in frozen transfer arm.

Of those randomized, 298 (97%) women in the elective freeze arm and 303 (98%) women in the fresh embryo transfer arm had an embryo transfer. Most embryo transfers (94.6% in frozen and 93.1% in fresh) involved embryos at blastocyst stage. In the elective freeze arm, embryo freezing was by vitrification at blastocyst stage in 88.1% cases. Almost all frozen embryo transfers were carried out in hormonally mediated cycles (206/223) (Table  I). Over 80% of women in both randomized groups received a single embryo; the others received two embryos, with the exception of one woman who had a triple embryo transfer.

In order to transfer 248 embryos, 280 had to be thawed, i.e. 88.6% were suitable to be transferred after being thawed. Three couples in the frozen transfer group did not have any embryos to transfer owing to the failure of all embryos to survive the freezing thawing process.

In the elective freeze group, the clinical characteristics pre-randomization (number of eggs, method of insemination, number of 2pn, number of good quality embryos on Day 3, cycle number and number of previous embryo transfers) were similar in the groups who complied with allocated intervention and those who did not (Supplementary Table SI). Median (IQR) of remaining embryos after first transfer were higher in those who complied compared to those who did not (3 (1–4) versus 1 (0–3)). This could partly be related to a lower proportion who had single embryo transfer (72.9% versus 88.6%) and a higher proportion that received blastocyst transfer (95.8% versus 88.1%) in the non-compliant group, leading to the use of more embryos at first transfer. More than 50% had at least one embryo remaining frozen after transfer in the non-compliant group.

**Figure 2.**
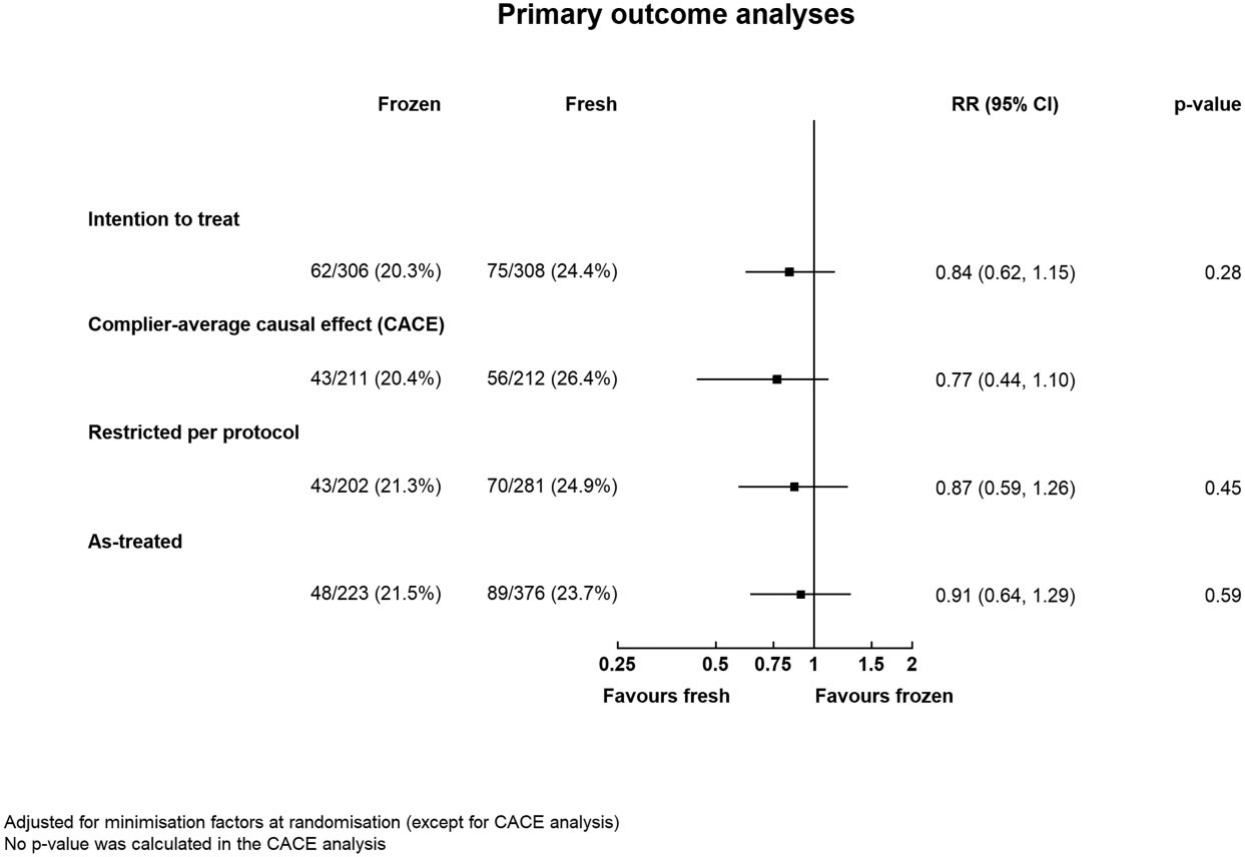
**Primary outcome (healthy baby rate) analyses.** RR, risk ratio.

ITT analysis showed that the healthy baby rate was 20.3% (62/307) in the elective freeze arm and 24.4% (75/309) in the fresh embryo transfer group (RR 0.84, 95% CI: 0.62 to 1.15) (Table  II) after first embryo transfer following randomization. The treatment effect (RR, 95% CI) was similar using a complier-average causal effect analysis {0.77 (0.44 to 1.10)}, a per-protocol analysis {0.87 (0.59 to 1.26)}, and an as-treated analysis {0.91 (0.64 to 1.29)} (Fig.  2). Within the elective freeze arm, the healthy baby rate was similar (21.3% versus 20.0%) between those who adhered to the allocated intervention and those who did not. There was no evidence of any interaction between treatment and subgroup in the healthy baby rate across all pre-specified subgroups: age of female partner (<35 or ≥35 years); previous embryo transfer performed (none or ≥1), or whether one or multiple embryos were transferred (Supplementary Fig. S1). It was not possible to perform subgroup analysis by cleavage versus blastocyst transfer and where female age was over 40 years owing to insufficient numbers.

**Table II:** Primary and secondary outcomes of the E-Freeze trial.

	Frozen embryo transfer (n = 307)	Fresh embryo transfer (n = 309)	Unadjusted risk ratio (95 or 99% CI)	Adjusted* risk ratio (95 or 99% CI)	*P*-value
**Primary outcome: singleton baby born at term with appropriate weight for gestation**	62 (20.3%)	75 (24.4%)	0.83 (0.62 to 1.12)	0.84 (0.62 to 1.15)	0.28
Missing	1	1			
**Measures of clinical effectiveness**					
**Live birth episode**	87 (28.3%)	106 (34.3%)	0.83 (0.61 to 1.13)	0.83 (0.65 to 1.06)	0.054
**Singleton baby**	85 (27.7%)	105 (34.0%)	0.81 (0.60 to 1.11)	0.82 (0.64 to 1.06)	0.048
**Clinical pregnancy**	104 (33.9%)	124 (40.1%)	0.84 (0.64 to 1.11)	0.85 (0.65 to 1.11)	0.11
**Maternal safety: ovarian hyperstimulation syndrome**	11 (3.6%)	25 (8.1%)	0.44 (0.18 to 1.10)	0.44 (0.15 to 1.30)	0.051
**Complications of pregnancy and delivery**					
**Miscarriage**	44 (14.3%)	40 (12.9%)	1.11 (0.66 to 1.87)	1.09 (0.72 to 1.66)	0.58
**Gestational diabetes mellitus**	4 (1.3%)	4 (1.3%)	1.00 (0.16 to 6.13)	NE	1.00
**Gestational diabetes mellitus in the clinically relevant population^†^**	4/87 (4.7%)	4/106 (3.9%)	1.21 (0.20 to 7.20)	NE	0.78
Missing	2	3			
**Hypertensive disorder**	8 (2.6%)	7 (2.3%)	1.15 (0.31 to 4.28)	NE	0.79
**Hypertensive disorder in the clinically relevant population^†^**	8/87 (9.4%)	7/106 (6.8%)	1.38 (0.39 to 4.97)	NE	0.51
Missing	2	3			
**Antepartum haemorrhage**	12 (3.9%)	13 (4.2%)	0.93 (0.34 to 2.55)	NE	0.85
**Antepartum haemorrhage in the clinically relevant population^†^**	11/87 (13.1%)	12/106 (11.7%)	1.12 (0.41 to 3.07)	NE	0.76
Missing	3	3			
**Preterm delivery (<37 completed weeks)**	9 (2.9%)	12 (3.9%)	0.75 (0.25 to 2.30)	NE	0.51
**Preterm delivery in the clinically relevant population^†^**	9/87 (10.3%)	12/106 (11.4%)	0.91 (0.31 to 2.65)	NE	0.81
Missing	0	1			
**Mode of delivery**					
Normal vaginal delivery	28 (9.2%)	38 (12.4%)	0.75 (0.41 to 1.37)	0.75 (0.54 to 1.05)	0.03
Instrumental vaginal delivery	20 (6.6%)	30 (9.8%)	0.68 (0.33 to 1.38)	0.69 (0.39 to 1.21)	0.09
Caesarean section	35 (11.6%)	36 (11.7%)	0.99 (0.55 to 1.75)	0.99 (0.67 to 1.47)	0.95
**Mode of delivery in the clinically relevant population^‡^**					
Normal vaginal delivery	28/89 (32.9%)	38/107 (36.2%)	0.91 (0.54 to 1.53)	0.92 (0.63 to 1.33)	0.56
Instrumental vaginal delivery	20/89 (23.5%)	30/107 (28.6%)	0.82 (0.43 to 1.56)	0.84 (0.56 to 1.27)	0.28
Caesarean section	37/89 (43.5%)	37/107 (35.2%)	1.24 (0.77 to 1.97)	1.21 (0.98 to 1.51)	0.02
Missing	4	2			
**Low birthweight (<2500 g at birth)**	7 (2.3%)	13 (4.2%)	0.54 (0.17 to 1.79)	NE	0.19
**Low birthweight in the clinically relevant population^‡^**	8/89 (9.1%)	14/107 (13.1%)	0.69 (0.24 to 2.05)	NE	0.39
Missing	1	0			
**High birthweight (>4000 g at birth)**	10 (3.3%)	10 (3.2%)	1.01 (0.33 to 3.14)	NE	0.98
**High birthweight in the clinically relevant population^‡^**	10/89 (11.4%)	10/107 (9.3%)	1.22 (0.41 to 3.62)	NE	0.64
Missing	1	0			
**Small for gestational age (<10th centile)**	8 (2.6%)	12 (3.9%)	0.67 (0.21 to 2.13)	NE	0.37
**Small for gestational age in the clinically relevant population^‡^**	9/89 (10.2%)	12/107 (11.3%)	0.90 (0.31 to 2.64)	NE	0.81
Missing	1	1			
**Large for gestational age (>90th centile)**	9 (2.9%)	10 (3.2%)	0.91 (0.28 to 2.90)	NE	0.83
**Large for gestational age in the clinically relevant population^‡^**	9/89 (10.2%)	10/107 (9.4%)	1.08 (0.35 to 3.33)	NE	0.85
Missing	1	1			
**Congenital anomaly/birth defect**	6 (2.0%)	7 (2.3%)	0.87 (0.21 to 3.57)	NE	0.79
**Congenital anomaly/birth defect in the clinically relevant population^‡^**	5/89 (5.7%)	5/107 (4.7%)	1.22 (0.25 to 5.95)	NE	0.75
Missing	2	1			

Data are presented as n (%), n/N (%), or n. CIs are 95% for the primary outcome and 99% for all secondary outcomes. *P*-values are for adjusted estimates when available, or unadjusted estimates otherwise.

NE, not estimable.

* Adjusted for woman’s age at ovarian stimulation, primary/secondary fertility, duration of infertility, method of insemination, number of previous egg collections and fertility clinic (as a random effect).

† Per total number of women with an ongoing pregnancy resulting in delivery who delivered.

‡ Per total number of babies born.

The risk of OHSS was 3.6% (11/307) in the elective freeze arm compared to 8.1% (25/309) in the fresh embryo transfer arm (RR 0.44, 99% CI: 0.15 to 1.30) (Table  II). The severity of ovarian hyperstimulation was only mild to moderate in the elective freeze group, whereas there were 6 cases (1.9%) of severe OHSS in the fresh embryo transfer group.

The live birth rate {28.3% versus 34.3%; RR, 99% CI: 0.83 (0.65 to 1.06)} and clinical pregnancy rates {33.9% versus 40.1%; RR, 99% CI: 0.85 (0.65 to 1.11)} were lower in the elective freeze arm, but there is no statistically significant difference (Table  II). The risk of miscarriage was similar in both groups (14.3% versus 12.9%, RR, 99% CI: 1.09, 0.72 to 1.66) when analysed by ITT or by clinically relevant denominator, i.e. per pregnancy {31.7% versus 26.0%; RR, 99% CI: 1.18 (0.76 to 1.84)}.

There was no evidence of a difference (RR, 99% CI) in the risk of gestational diabetes mellitus {4.7% versus 3.9%; RR, 99% CI: 1.21 (0.20 to 7.20)} or hypertensive disorder in pregnancies {9.4% versus 6.8%; RR, 99% CI: 1.38 (0.39 to 4.97)} in pregnancies in the elective freeze arm compared to fresh embryo transfer arm. There were no cases of eclampsia in the trial. There were five cases of pre-eclampsia (5.9%) in pregnancies in the elective freeze group compared to 1 (1%) in the fresh embryo transfer group. The was no evidence of a difference in the risk of antepartum haemorrhage {13.1% versus 11.7%; RR, 99% CI: 1.12 (0.41 to 3.07)} and preterm delivery {10.3% versus 11.4%; RR, 99% CI: 0.91 (0.31 to 2.65)} in the elective freeze group compared to fresh embryo transfer group.

A total of 196 babies were born (89 in the elective freeze arm versus in 107 in the fresh embryo transfer arm). One-third of women (32.9% versus 36.2%) had normal vaginal delivery (RR, 99% CI: 0.92, 0.63 to 1.33); 23.5% versus 28.6% had an instrumental vaginal delivery (RR, 99% CI: 0.84, 0.56 to 1.27); and 43.5% versus 35.2% had Caesarean section {RR, 99% CI: 1.21 (0.98 to 1.51)} in the elective freeze versus the fresh embryo transfer arm, respectively.

There was no evidence of a significant difference in the risk (RR: 99% CI) of having a low birthweight {9.1% versus 13.1%; RR, 99% CI: 0.69 (0.24 to 2.05)}, high birthweight {11.4% versus 9.3%; RR, 99% CI: 1.22 (0.41 to 3.62)}, SGA {10.2% versus 11.3% RR, 99% CI: 0.90 (0.31 to 2.64)} or a LGA baby {10.2% versus 9.4%; RR, 99% CI: 1.08 (0.35 to 3.33)} in babies born in elective freeze arm when compared with fresh embryo transfer arm. There was no evidence of a difference in the rate of congenital anomaly either (5.7% versus 4.7%) with RR, 99% CI as 1.22 (0.25 to 5.95). There was one neonatal death in the elective freeze arm and none in fresh embryo transfer group.

### Economic analysis

Post-randomization IVF-related treatment costs were higher in the elective freeze than fresh transfer arm (£1538 versus £1216) owing to the higher number of pre-embryo transfer monitoring visits and transvaginal ultrasound scans. Costs of OHSS, however, were higher in the fresh transfer arm owing to the higher incidence of this complication (8.1% versus 3.6%). The mean cost (inclusive of treatment and OHSS management costs) was higher (+£170, 95% CI: 67 to 289) but the healthy baby rate (−0.039, 95% CI −0.101 to 0.027) and live birth rate (−0.06, 95% CI: −0.127 to 0.020) were lower in the elective freeze than fresh transfer arm, although these differences were not statistically significant (Supplementary Table SII). Using bootstrap resampling to characterize the uncertainty around the estimated joint difference in costs and effects (Supplementary Fig. S2), electively freezing all suitable embryos had a low chance of being considered cost-effective at all WTP thresholds. The magnitude and statistical significance of the mean cost-difference was sensitive to the unit cost applied to transvaginal ultrasound scans (Supplementary Table SIII), but the probability of cost-effectiveness remained low for the elective freeze approach (Supplementary Fig. S3).

The cost for pregnancy care was similar between groups, and fresh embryo transfer retained the higher probability of being cost-effective from the UK perspective above a WTP threshold of £1921 per additional healthy live birth (Supplementary Table SIII and Fig. S3).

## Discussion

The results of this study, despite limited sample size, showed that a policy of electively freezing all suitable embryos followed by thawed frozen embryo transfer did not increase the chance of having a healthy baby after first embryo transfer but was significantly more expensive from the UK perspective. The risk of OHSS was not reduced by an elective freeze policy. There was no evidence of a statistically significant difference in live birth, clinical pregnancy and miscarriage rates in those who were randomized. A high level of non-adherence in couples randomized to the elective freeze is suggestive of a preference for fresh embryo transfer.

This is the first UK trial comparing fresh embryo transfer with a policy of electively freezing all suitable embryos followed by subsequent frozen embryo transfer. E-Freeze was a pragmatic trial and the participants were recruited from a total of 18 NHS and private clinics, as 70% of IVF treatment in the UK is self-funded by couples. Withdrawal from the trial was minimal and data collection was almost complete. Despite not reaching the original planned sample size of 1086, it still represents the largest trial outside Asia to address this question along with detailed health economic analysis.

This trial did not recruit to the initial planned numbers, however, in view of the trends identified in the data (higher clinical pregnancy rate and live birth rate in fresh embryo transfer but not statistically significant) a statistically significant change in direction of the results would be unlikely even if 1086 couples were recruited.

We have not reported on cumulative healthy baby rate in this manuscript as that is a follow-up study. It is well known that cumulative outcomes are more important than outcomes after single embryo transfer. We will be reporting on them in the near future.

The reported difference in costs is only valid for the UK and therefore, this money-saving benefit may not be as significant in other clinics/countries with different characteristics/protocols.

The significant drop in numbers of participants between consent and randomization mainly resulted from the absence of three good quality embryos in a large proportion of recruited couples. This was primarily caused by the broad inclusion criteria, which did not exclude those who were less likely to have a good prognosis. There was high non-adherence to the allocated intervention in the elective freeze arm, despite minimal delay between randomization and delivery of the intervention (embryo transfer) and sufficient time between consent and randomization to ensure a well-informed consent process. The most common reason for non-adherence was personal choice owing to a strong preference for fresh embryo transfer. This is interesting as the studies exploring the intentions of couples (Stormlund *et al.*, 2019; Abdulrahim *et al.*, 2021) suggest that they do not prefer fresh over elective freezing when hypothetical scenarios are given. When the benefits of a freeze-all strategy were explained in detail to the participants there was no preference whatsoever. However, from this trial, it is clear that intentions do not always translate into real practice. There could be important cultural influence as well as in preference towards the fresh embryo transfer, which we could not elicit in this study.

When the trial was designed embryo transfer was usually performed on Day 3 but this changed during the trial to Day 5. This created a slightly longer gap between randomization (Day 3) and intervention (Day 5), which allowed clinicians and participants to change their minds in favour of fresh embryo transfer. Limited public funding for IVF and no compensation (e.g. free IVF cycle) for those participating in the trial as well-participant preference may have contributed to non-adherence. The analyses by complier average casual effect, per-protocol and as-treated did not have a noteworthy impact on the results, suggesting that non-adherence is unlikely to have altered the overall interpretation of the findings of this trial. Clinical characteristics were also similar between those who complied and those who did not comply with allocated intervention in elective freeze group, hence it was down to participant’s own choice.

During the conduct of E-Freeze, five large trials (Shi *et al.*, 2018; Vuong *et al.*, 2018; Wei *et al.*, 2019; Stormlund *et al.*, 2020; Wong *et al.*, 2021) were published on normal responders. Despite different designs, with randomization at various points in the IVF treatment, the overall results are very similar to E-Freeze. None of these other trials reported on healthy baby rate, hence data on this outcome could not be compared. Since all complications in pregnancy and delivery have an impact on the short- and long-term health of an individual, E-Freeze was unique in taking a holistic view of efficacy and safety, evaluating the healthy baby rate and not just live birth. We also reported on details of obstetrics and perinatal outcomes.

Our trial did not show a statistical difference in OHSS between the two arms. One of the reasons could be that most patients received HCG as randomization was not until Day 3 after fertilization. However, others who have randomized at the start of stimulation also showed no difference in the risk of OHSS (Stormlund *et al.*, 2020). This could be related to the low number of cases in each trial.

In the aftermath of the coronavirus disease 2019 (COVID-19) pandemic, national and international guidance (American Society of Reproductive Medicine, ESHRE and British Fertility Society) has tended to recommend a low threshold for freezing all embryos, as a precautionary measure (COVID-19 and ART (eshre.eu)). With the increasingly widespread practice of elective freeze in preference to fresh embryo transfer across IVF clinics, this trial provides timely evidence, though limited by not reaching full sample size, for practitioners to re-evaluate this approach in the absence of a strong clinical indication, such as significant risk of OHSS.

For elective freezing of all suitable embryos to be as accepted as the default strategy for all, it must show clinical and cost-effectiveness especially as this involves a delay in getting pregnant, extra clinic activity and additional visits for patients. There was a clear consensus from clinicians and scientists prior to this trial that a policy of electively freezing all suitable embryos should only be used if it improves the absolute healthy baby rate by at least 8%.

A Cochrane review (Zaat *et al.*, 2021) has suggested that there is moderate quality evidence that elective freeze policy is not better than fresh embryo transfer in terms of cumulative live birth rate and ongoing pregnancy rates. However, in the absence of individual participant data, it was not possible to conduct meaningful subgroup analyses based on important characteristics such as maternal age, embryo number and quality, hence the debate continues. Meta-analyses of observational data have also shown that singletons born as a result of frozen embryo transfer are at lower risk of preterm delivery and SGA but at higher risk of LGA and pre-eclampsia (Maheshwari *et al.*, 2018). Meta-analysis of RCTs (Zaat *et al.*, 2021) confirmed a higher risk of LGA and hypertensive disorders but failed to show a difference in preterm delivery and SGA. Thus, despite the availability of randomized data from over 5000 patients, there is no consensus on the clinical and cost-effectiveness of a blanket policy of electively freezing all suitable embryos. The available RCTs are powered for live birth rates and are unable to comment on the comparative benefits and risks of fresh versus frozen embryo transfer with respect to less common outcomes and in key subgroups. The effectiveness of elective freezing of all suitable embryos followed by frozen embryo transfer may vary by maternal age, number of eggs obtained, number of embryos, stage of embryo transfer and type of freezing: sub-group analyses may help to identify the couples undergoing IVF for whom this strategy is particularly effective.

Rather than investing additional time and resources in further RCTs, we believe that an individual participant data meta-analysis (IPD-MA) offers a more efficient and cost-effective way of addressing this evidence gap. An IPD-MA approach (Riley *et al.*, 2010) will allow researchers to estimate the incidence of clinically important but less common pregnancy and neonatal complications and help to develop a personalized approach based on individualized prediction of success rates associated with fresh versus frozen embryo transfer.

In conclusion, the results of this multicentre pragmatic RCT do not support a change to a universal elective freeze policy on grounds of clinical or cost-effectiveness although the results were limited by not reaching full sample size as well as non-adherence.

## Data availability

Data will be shared in accordance with the National Perinatal Epidemiology Unit Data Sharing policy. Requests for access to the data will be considered by the National Perinatal Epidemiology Unit Data Sharing committee. Access to anonymized data can be requested from general@npeu.ox.ac.uk. The trial protocol, statistical analysis plan and other study documents are also available through this route.

## Supplementary Material

deab279_Supplementary_Figure_S1

deab279_Supplementary_Figure_S2

deab279_Supplementary_Figure_S3

deab279_Supplementary_Table_S1

deab279_Supplementary_Table_S2

deab279_Supplementary_Table_S3
