# Supplementary material for: Elective freezing of embryos versus fresh embryo transfer in IVF: a multicentre randomized controlled trial in the UK (E-Freeze)
Source: Hum Reprod. 2022 Jan 6;37(3):476–87. doi: 10.1093/humrep/deab279 (PMC9206534; doi:10.1093/humrep/deab279)
Supplement: deab279_Supplementary_Figure_S3 [file deab279_supplementary_figure_s3.pdf]

Human Reproduction, pp. 1–1, 2022
https://doi.org/10.1093/humrep/deab279

human reproduction

SUPPLEMENTARY DATA

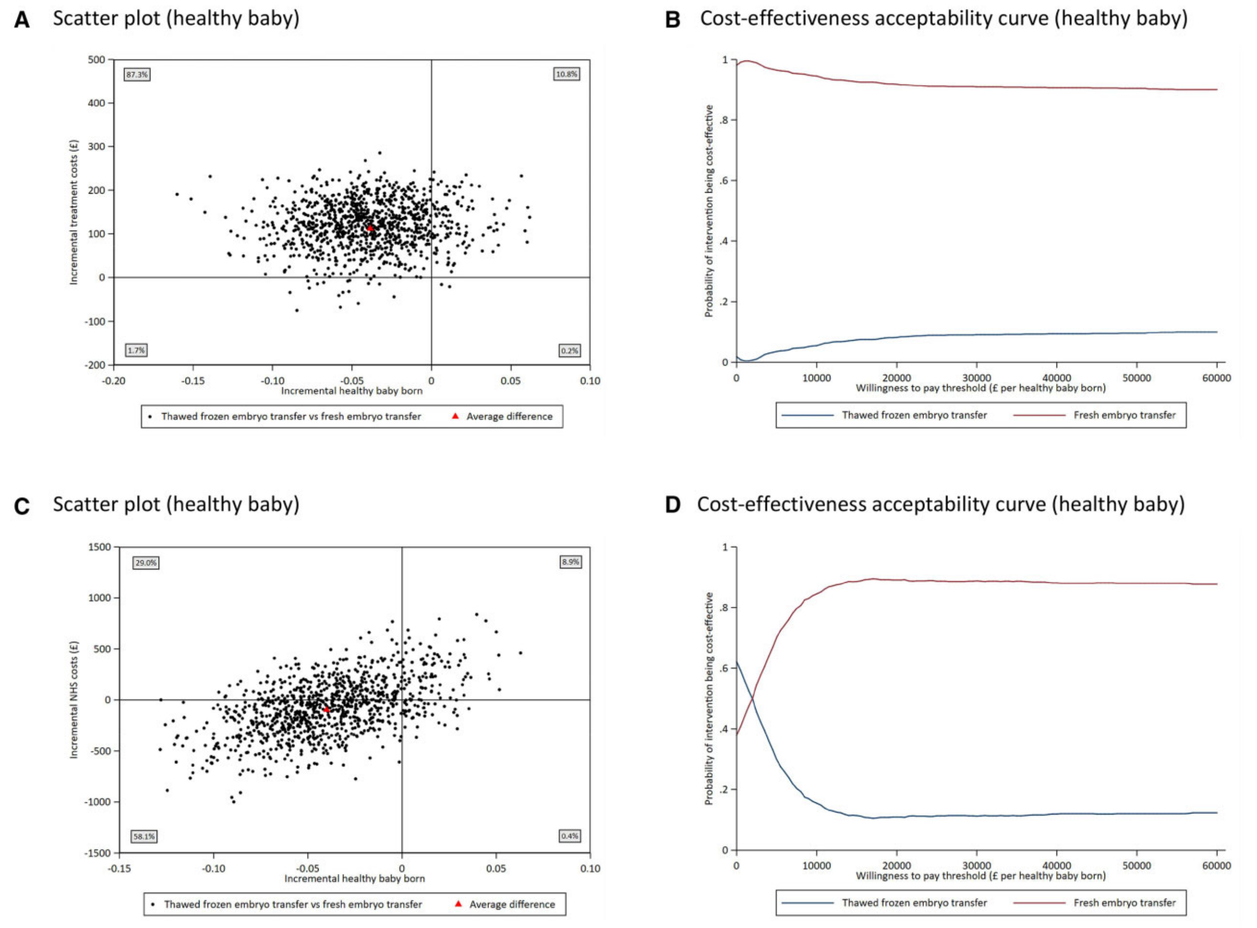

Supplementary Figure S3. Sensitivity analysis showing the scatter plot and acceptability curve for the incremental cost per health baby. (**A** and **B**) Including transvaginal scan for monitoring. (**C** and **D**) Antenatal care and delivery costs.
