# Supplementary material for: Elective freezing of embryos versus fresh embryo transfer in IVF: a multicentre randomized controlled trial in the UK (E-Freeze)
Source: Hum Reprod. 2022 Jan 6;37(3):476–87. doi: 10.1093/humrep/deab279 (PMC9206534; doi:10.1093/humrep/deab279)
Supplement: deab279_Supplementary_Table_S1 [file deab279_supplementary_table_s1.pdf]

Human Reproduction, pp. 1–1, 2022
https://doi.org/10.1093/humrep/deab279

human reproduction

SUPPLEMENTARY DATA

Supplementary Table SI **Baseline characteristics in those allocated to elective freeze arm for those who complied with the allocated intervention and those who did not.**

	Complied: received frozen embryo transfer (n = 202)	Did not comply: received fresh embryo transfer (n = 96)
**At trial entry**		
**Woman's age at ovarian stimulation (years)***	35 (3.5)	33.9 (4.1)
**Non-smoker**	179 (88.6)	92 (95.8)
**Woman's BMI (kg/m^2)†**	23.9 (3.3)	24.5 (3.6)
**Primary infertility***	156 (77.2)	75 (78.1)
**Primary cause of infertility**		
Ovulatory	24 (11.9)	16 (16.7)
Tubal	20 (9.9)	8 (8.3)
Endometriosis	7 (3.5)	5 (5.2)
Unexplained	83 (41.1)	33 (34.4)
Male	64 (31.7)	34 (35.4)
Other	4 (2.0)	0
**Duration of infertility (months)***	36 (24 to 48)	36 (25 to 48)
**Total stimulation dose of FSH (IU)**	2612.5 (1171.2)	2363.3 (1424.2)
**Total number of eggs collected**	13 [9 to 16]	12 [9 to 16]
**Method of insemination—IVF***	108 (53.5%)	47 (49%)
**Good quality embryos on Day 3***	5 (4 to 8)	5 (3 to 6)
**During treatment**		
**Stage of embryo at transfer—blastocyst**	178 (88.1%)	92 (95.8%)
**Single embryo transfer**	179 (88.6%)	70 (72.9%)
**Number of remaining frozen embryos after transfer (median (IQR))**	3 (1 to 4)	1 (0 to 3)
0	28 (13.9%)	40 (41.7%)
1	33(16.3%)	13 (13.5%)
2	40 (19.8%)	15 (15.6%)
≥3	101 (50.0%)	28 (29.2%)

Data are presented as mean (SD), median (IQR), N or n/N (%).
*Minimization factor.
†One observation missing.
