# Supplementary material for: Elective freezing of embryos versus fresh embryo transfer in IVF: a multicentre randomized controlled trial in the UK (E-Freeze)
Source: Hum Reprod. 2022 Jan 6;37(3):476–87. doi: 10.1093/humrep/deab279 (PMC9206534; doi:10.1093/humrep/deab279)
Supplement: deab279_Supplementary_Table_S2 [file deab279_supplementary_table_s2.pdf]

Human Reproduction, pp. 1–1, 2022
https://doi.org/10.1093/humrep/deab279

human reproduction

SUPPLEMENTARY DATA

Supplementary Table SII **Trial-based incremental cost per healthy baby and live birth (National Health Service perspective).**

	Total cost (£) mean (95% CI)	Incremental cost (£) mean (95% CI)	Total effect mean (95% CI)	Incremental effect mean (95% CI)	Incremental cost-effectiveness ratio
**Treatment costs, healthy baby**					
Fresh embryo transfer	1402 (1279 to 1516)		0.242 (0.197 to 0.294)		
Freeze all	1572 (1518 to 1641)	170 (61 to 284)	0.204 (0.160 to 0.246)	−0.039 (−0.104 to 0.023)	Dominated
**Treatment costs, live birth**					
Fresh embryo transfer	1401 (1297 to 1517)		0.341 (0.289 to 0.397)		
Freeze all	1572 (1516 to 1642)	170 (67 to 289)	0.285 (0.235 to 0.331)	−0.057 (−0.138 to 0.013)	Dominated
