# Supplementary material for: Elective freezing of embryos versus fresh embryo transfer in IVF: a multicentre randomized controlled trial in the UK (E-Freeze)
Source: Hum Reprod. 2022 Jan 6;37(3):476–87. doi: 10.1093/humrep/deab279 (PMC9206534; doi:10.1093/humrep/deab279)
Supplement: deab279_Supplementary_Table_S3 [file deab279_supplementary_table_s3.pdf]

Human Reproduction, pp. 1–1, 2022
https://doi.org/10.1093/humrep/deab279

human reproduction

SUPPLEMENTARY DATA

Supplementary Table SIII **Within-trial sensitivity analysis of incremental cost per healthy baby.**

	Total cost (£) mean (95% CI)	Incremental cost (£) mean (95% CI)	Total effect mean (95% CI)	Incremental effect mean (95% CI)	Incremental Cost-effectiveness ratio
**Assuming the transvaginal scan cost was inclusive of a monitoring visit cost**					
Fresh embryo transfer	1397 (1292 to 1510)		0.242 (0.197 to 0.294)		
Freeze all	1509 (1461 to 1571)	112 (5 to 222)	0.204 (0.160 to 0.246)	−0.039 (−0.104 to 0.023)	Dominated
**Using the lower ultrasound scan cost (£53) to cost transvaginal ultrasound scans**					
Fresh embryo transfer	1393 (1289 to 1504)		0.242 (0.197 to 0.294)		
Freeze all	1443 (1401 to 1498)	50 (−56 to 157)	0.204 (0.160 to 0.246)	−0.039 (−0.104 to 0.023)	Dominated
**National Health Service costs inclusive of antenatal and delivery care[a]**					
Fresh embryo transfer	3545 (3138 to 3960)		0.232 (0.189 to 0.281)		
Freeze all	3469 (3102 to 3869)	−75 (−623 to 461)	0.193 (0.151 to 0.237)	−0.039 (−0.101 to 0.027)	1921

[a]Antenatal or delivery costs could not be determined for 11 patients (analysis based on 605 complete cases).
